# SNAREs Interact with Retinal Degeneration Slow and Rod Outer Segment Membrane Protein-1 during Conventional and Unconventional Outer Segment Targeting

**DOI:** 10.1371/journal.pone.0138508

**Published:** 2015-09-25

**Authors:** Rahel Zulliger, Shannon M. Conley, Maggie L. Mwoyosvi, Michael W. Stuck, Seifollah Azadi, Muna I. Naash

**Affiliations:** Department of Cell Biology, University of Oklahoma Health Sciences Center, Oklahoma City, Oklahoma, 73104, United States of America; Cedars-Sinai Medical Center; UCLA School of Medicine, UNITED STATES

## Abstract

Mutations in the photoreceptor protein peripherin-2 (also known as RDS) cause severe retinal degeneration. RDS and its homolog ROM-1 (rod outer segment protein 1) are synthesized in the inner segment and then trafficked into the outer segment where they function in tetramers and covalently linked larger complexes. Our goal is to identify binding partners of RDS and ROM-1 that may be involved in their biosynthetic pathway or in their function in the photoreceptor outer segment (OS). Here we utilize several methods including mass spectrometry after affinity purification, *in vitro* co-expression followed by pull-down, *in vivo* pull-down from mouse retinas, and proximity ligation assay to identify and confirm the SNARE proteins Syntaxin 3B and SNAP-25 as novel binding partners of RDS and ROM-1. We show that both covalently linked and non-covalently linked RDS complexes interact with Syntaxin 3B. RDS in the mouse is trafficked from the inner segment to the outer segment by both conventional (i.e., Golgi dependent) and unconventional secretory pathways, and RDS from both pathways interacts with Syntaxin3B. Syntaxin 3B and SNAP-25 are enriched in the inner segment (compared to the outer segment) suggesting that the interaction with RDS/ROM-1 occurs in the inner segment. Syntaxin 3B and SNAP-25 are involved in mediating fusion of vesicles carrying other outer segment proteins during outer segment targeting, so could be involved in the trafficking of RDS/ROM-1.

## Introduction

Peripherin-2 also known as RDS (retinal degeneration slow) is an integral membrane tetraspanin protein found in the rim region of the outer segment (OS) discs in rod and cone photoreceptors [1]. The OS is a modified cilium and proteins found in the OS are synthesized in an adjacent, but distinct, cellular compartment called the inner segment (IS). RDS is a structural protein required for the formation of OSs, and mice homozygous for a naturally occurring RDS null allele (*rds* ^*-/-*^ also known as *rd2*) do not form OSs and exhibit progressive photoreceptor degeneration [2]. In addition to its role as a structural protein, RDS also has membrane fusogenic activity [3, 4], and the ability to induce membrane curvature [5]. Multiple different RDS mutations cause widely varying forms of rod- and cone- dominant retinal diseases including autosomal dominant retinitis pigmentosa and macular dystrophy in patients [6].

In the disc rim, RDS is tightly associated with ROM-1 (rod outer segment protein 1) a non-glycosylated homolog with high structural similarity to RDS [7]. After synthesis, RDS and ROM-1 form non-covalently linked homo- and heterotetramers. Subsequently, these core complexes covalently link into higher-order complexes which are found in the OS [8, 9]. Assembly of these higher-order complexes involves the formation of an intermolecular disulfide bond mediated by C150. C150 is one of seven cysteines found in the second extracellular/intradiscal (D2) loop of RDS and is the only D2 cysteine not involved in intramolecular interactions [10, 11]. The importance of RDS/ROM-1 complex assembly is underscored by the observation that many disease-causing-mutations alter complex formation [12, 13] and that OSs fail to form in the absence of higher-order complexes (i.e. in the C150S transgenic mouse on the *rds* ^*-/-*^ background) [10, 14].

Both our past research and the widely varying disease phenotypes in patients suggest that RDS may function differently in rod vs. cone photoreceptors [6, 13, 15]. Though the reasons for this are unclear, we have hypothesized that as yet unidentified binding partners of RDS/ROM-1 complexes may play a role in the function of these proteins in photoreceptors. These binding partners could interact with RDS/ROM-1 in a transient fashion, e.g. during OS targeting or at the base of the OS during disc assembly, or at the tip of the OS during the OS phagocytosis process. Two known binding partners of RDS fall into this category, calmodulin and melanoregulin, both of which are thought to regulate the fusogenic capability of RDS [16, 17]. Alternatively, interactions between RDS and its binding partners could be involved in more long-term interactions in the OS, possibly to stabilize or regulate the structure of the OS. One known RDS binding partner, the GARP/beta subunit of the rod cyclic nucleotide gated channel (CNGβ) likely falls into this second category, and OS interactions between RDS and GARP/ CNGβ [18, 19] may be involved in linking adjacent OS rims or linking rims with the OS plasma membrane.

Here, our goal was to identify novel RDS/ROM-1 interacting partners. Three members of the SNARE family were identified, of which two (Syn3B and SNAP-25) were subsequently validated. These SNARE proteins are known to be involved in the trafficking of other OS integral membrane proteins such as rhodopsin [20], so it is possible they may play a similar role with RDS/ROM-1.

## Materials and Methods

### Animals

All animal handling, procedures, and maintenance were approved by the University of Oklahoma Health Sciences Center Institutional Animal Care and Use Committee, and followed guidelines set forth by the Association for Research in Vision and Ophthalmology. Animals were reared on a 12H L/D cycle with maximum light intensity of ~30 lux. All mice were negative for the *rd8* allele. Retinas from the following mouse strains were used: WT (Jackson Laboratories, Bar Harbor, ME), *rds* ^*-/-*^ (originally shared by Dr. Neeraj Agarwal, University of North Texas Health Science Center, Fort Worth, TX), *rom1* ^*-/-*^ (shared by Dr. Roderick McInnes, University of Toronto, Toronto, Canada), *nrl* ^*-/-*^ (shared by Dr. Anand Swaroop, National Eye Institute, Bethesda, MD), knock-in lines carrying a single point mutation in RDS (*rds* ^*Y141C/Y141C*^ [12], *rds* ^*C150S/C150S*^ and *rds* ^*N229S/N229S*^, generated for us by inGenious Targeting Laboratory, Ronkonkoma, NY), and previously characterized transgenic mouse lines expressing mutant RDS on the *rds* ^*-/-*^ or *rds* ^*-/-*^ */nrl* ^*-/-*^ background (R172W/*rds* ^*-/-*^, R172W/*rds* ^*-/-*^ */nrl* ^*-/-*^ [13]). Animals were euthanized at post-natal day (P) 30 for the current study and retinas were harvested as described previously [21].

### Antibodies

Multiple antibodies for each protein were used and are indicated in the figure legends. Antibodies are summarized in **Table 1.** For RDS, we used RDS-CT (rabbit polyclonal [21]) at 1:1,000 for western blot [WB] and immunofluorescence [IF], RDS 2E7 (mouse monoclonal, generated by Precision Antibody) and RDS 2B7 (mouse monoclonal [13]), both used at 1:1,000 for WB and IF, and RDS-PCT1 (sheep polyclonal generously shared with us by Dr. Vadim Arshavsky, Duke University, Chapel Hill, NC) at 1:1,000 for IF. For ROM-1, we used anti-ROM-1 2H5 (mouse monoclonal [13]) at 1:5 for WB and 1:3 for IF, and ROM1-CT (rabbit polyclonal [21]) at 1:1,000 for IF and WB, and ROM1-CT1 (sheep polyclonal generously shared with us by Dr. Vadim Arshavsky, Duke University, Chapel Hill, NC) at 1:1,000 for IF. For Syn3B, we used anti-Syn3B-SySy (rabbit polyclonal, cat# 110 032, Synaptic Systems, Goettingen, Germany) at 1:1,000, Syn3B-478 and Syn3B-529 (both rabbit polyclonal, a kind gift from Dr. Roger Janz, University of Texas Medical School, Houston, TX) at 1:1,000, and Syn3B 12E5. Syn3B 12E5 is a new mouse monoclonal generated in house and used at 1:1,000 for WB (**S1 Fig**). We also used anti-SNAP-25 (cat# SMI-81R, mouse monoclonal, Covance, Princeton, NJ) at 1:1,000, anti-flag DDK (cat# TA50011, mouse monoclonal, Origene, Rockville, MD) at 1:1,000, anti-flag DYKDDDDK (cat# 2368, rabbit polyclonal, Cell Signaling Technology, Danvers, MA) at 1:1,000, anti-VAMP2 (mouse monoclonal, cat# 104 211, Synaptic Systems, Goettingen, Germany) at 1:10,000 for WB and 1:1,000 for IF, and anti-Cytochrome C (rabbit polyclonal, cat# 4272, Cell Signaling, Danvers, MA) at 1:100 for IF, anti-Na^+^K^+^ATPase (mouse monoclonal, a5, Developmental Studies Hybridoma Bank, University of Iowa, Iowa City, IA) used at 1:500 for IF, and anti-prominin-1 (rat, clone 13A4, eBiosciences, San Diego, CA) used at 1:1,000 for IF. The a5 monoclonal antibody was developed by D.M. Fambrough, and was obtained from the Developmental Studies Hybridoma Bank, created by the NICHD of the NIH and maintained at The University of Iowa, Department of Biology, Iowa City, IA 52242. Anti-rhodopsin (1D4, a monoclonal antibody) and anti-CNGB1/GARP (4B1 a monoclonal antibody) kind gifts from Dr. Robert Molday, University of British Columbia, Vancouver, Canada, and anti-arrestin 1 (mouse, clone E-3, Santa Cruz Biotechnology, Dallas, TX) were used at 1:1,000 for WB. Anti-β-actin HRP antibody (AC-15 monoclonal antibody, Sigma-Aldrich, St. Louis, MO) was used at 1:50,000 for WB. Secondary antibodies were goat anti-mouse HRP and goat anti-rabbit HRP (KPL, Gaithersburg, MD), used at 1:25,000 for WB, and donkey anti-rabbit Alexafluor 555, donkey anti-mouse Alexafluor 488, donkey anti-sheep Alexafluor 488 and donkey anti-rat Alexafluor 594 (Life Technologies, Grand Island, NY), both used at 1:1,000 for IF.

**Table 1:** Antibodies Used.

Antigen	Species	Clone	Source	Cat. #	Ref.
**RDS**	Rabbit	RDS-CT	In house		[21]
	Mouse	2E7	Precision Antibody		
	Mouse	2B7	Precision Antibody		[13]
	Sheep	RDS-PCT1	V. Arshavsky		
**ROM-1**	Mouse	2H5	In house		[13]
	Rabbit	ROM1-CT	In house		
	Sheep	ROM1-CT1	V. Arshavsky		[21]
**Syn3B**	Rabbit		Synaptic Systems	110 032	
	Mouse	12E5	In House		
	Rabbit	478	R. Janz		[22]
	Rabbit	529	R. Janz		
**SNAP-25**	Mouse		Covance	SMI-81R	
**Flag DDK**	Mouse	OTI4C5	Origene	TA50011	
**Flag DYKDDDDK**	Rabbit		Cell Signaling	2368	
**VAMP-2**	Mouse	69.1	Synaptic Systems	104 211	
**Cytochrome C**	Rabbit		Cell Signaling	4272	
**Na^+^/K^+^-ATPase**	Mouse	A5	Hybridoma Bank		
**Prominin-1**	Rat	13A4	eBiosciences	14–1331	
**Rhodopsin**	Mouse	1D4	R. Molday		[23]
**Arrestin 1**	Mouse	E-3	Santa Cruz Biotechnology	sc-166383	
**GARP/CNGB1**	Mouse	4B1	R. Molday		[18]
**Actin**	Mouse	AC-15	Sigma-Aldrich	A3854	
**Rabbit IgG HRP**	Goat		KPL	074–1506	
**Mouse IgG HRP**	Goat		KPL	074–1807	
**Rabbit IgG 555**	Donkey		Life Technologies	A-31572	
**Mouse IgG 488**	Donkey		Life Technologies	R37114	
**Sheep IgG 488**	Donkey		Life Technologies	A-11015	
**Rat IgG 594**	Donkey		Life Technologies	A-21209	

### Proteomics

For LC-MS, 40 mouse retinas from either wild-type (WT) or *rds* ^*-/-*^ mice were homogenized by passing through a series of needles ranging from 18G-26G, after suspending in homogenization buffer containing 20 mM Tris-HCl, pH 7.2, 100 mM NaCl, 1 mM MgCl_2_ and protease inhibitor (Roche Applied Sciences, Indianapolis, IN). Sucrose was added to a final concentration of 12%. The extract was layered on top of a 50%/60% sucrose gradient and centrifuged at 43,000 x g for 40 min. The pellet was resuspended in homogenization buffer to dilute the sucrose and subsequently centrifuged at 32,000 x g. The pellet was then solubilized in buffer containing 20mM HEPES, pH 7.5, 150mM NaCl, 5mM CHAPS, 0.55mM DTT and protease inhibitor (Roche Applied Sciences, Indianapolis, IN) for 1h at 4°C. The protein extract was subjected to immunoprecipitation (IP) with antibodies against RDS, ROM-1, Syn3B, or IgG (control). For RDS (2E7) and ROM-1 (2H5), antibodies were chemically linked to sepharose 2B beads and extracts were incubated with beads for 1 h at 4°C. For the Syn3B and IgG, the protein was incubated with the antibody (Synaptic Systems, Goettingen, Germany) and protein A beads overnight at 4°C. Elution was performed with a 1% SDS solution and was separated on SDS-PAGE. The proteins were subjected to trypsin digestion in-gel (250 ng), extracted from the gel and freeze dried. Analysis of the protein fragments was carried out by LC-MS (ESI-TRAP) by the University of Rochester Proteomics Center. Analysis of the peptide fragments was performed using Scaffold 4 Proteomics software (Proteome Software Inc., Portland OR). The peptide fragments were identified by using a subset of the NCBInr_20130830 database restricted to Mus musculus and search engines Mascot and X!Tandem. Proteins were included in subsequent analysis if their identity was established at a probability level of 95% or higher and at least one peptide had been identified (Probability model Peptide prophet with Delta mass correction) [24, 25]. In addition, only proteins with a mascot score higher than 37 were considered as identified.

### Vectors and Transient Transfections

Vectors containing WT ROM-1 cDNA, WT RDS cDNA, or RDS cDNA with the C150S mutation under the control of the CMV promoter were generated by site-specific mutagenesis of the previously characterized pCDNA3.1-RDS/ROM-1 vectors [26]. We acquired the pCMV6-syntaxin 3B and pCMV6-VAMP2-myc-DDK (Origene, Rockville, MD), which both contain a CMV promoter and the cDNA sequence for Syn3B or VAMP2, respectively. The VAMP2 sequence was c-terminally tagged with flag (DDK) and Myc. The cDNA sequence for SNAP-25 was acquired in pCMV-SPORT6-SNAP-25 with a CMV promoter (Thermo Scientific, Pittsburgh, PA). HEK293 cells grown in DMEM (Life Technologies, Grand Island, NY) with antibiotic/antimycotic solution (Corning Cellgro, Corning, NY) and 5% fetal bovine serum, were transfected with equimolar amounts of the indicated vectors (~3.3μg/million cells, 1x10^6^ cells per 60.1 cm^2^ petri dish, covered with 2ml of medium). The DNA was added to 500 μl of a 250 mM CaCl_2_ solution and an equal amount of 2x BBS (50 mM BES, 280 mM NaCl, 1.4 mM Na_2_HPO_4_, pH 6.96) was added while vortexing at 1700 rpm. The transfection mixture was incubated at room temperature for 20 min and then added to the cells. The medium was replaced after an overnight incubation, and cells were harvested after 48 h.

### Immunoprecipitation (IP)

Retinas or cell extracts were quickly sonicated in 1x phosphate buffered saline (PBS, 137 mM NaCl, 2.68 mM KCl, 1.47 mM KH_2_PO_4_, 8.1 Na_2_HPO_4_, pH 7.0) with 5 mM EDTA, 5 mg/ml NEM (N-ethylmaleimide) and protease inhibitor (Roche Applied Sciences, Indianapolis, IN). Either 2% Triton X-100 and 2.5% glycerol or 10 mM CHAPS was added as indicated in the results. The retinal extract was solubilized at 4°C for 1 hour on a rocker and then centrifuged at 18,000 xg for 10 minutes. 100 μg of retinal extract (diluted to 0.5 μg/μl in 200 μl) (or 300 μg for *rds* ^*Y141C/Y141C*^ and R172W/*rds* ^*-/-*^ and 500 μg for *rds* ^*C150/C150S*^ and *rds* ^*-/-*^ to adjust for low levels of RDS/ROM-1 in these lines) or 50 μg cell extract (diluted to 0.25 μg/μl in 200 μl) was incubated for 2 h at 4°C in the presence of RDS-CT, ROM-1-CT, or Syn3B-478. Subsequently, protein A sepharose beads (GE Healthcare, Pittsburgh, PA) were added for 1 h. The beads were washed and eluted with 50 μl of a 1:1 mixture of the extraction buffer and 4x Laemmli buffer containing 400mM DTT. For non-reducing gels, the same buffer was used, but without DTT. For IPs carried out using retinal extract, 7.5 μg (15 μl) of protein was loaded for the input and an equal volume of the unbound fraction (15 μl, flow through). We loaded 65% of the bound fraction. Western blots were performed using standard protocols and detection was performed with HRP substrate (prepared after [27]) on a ChemiDoc^TM^ MP imager (Bio-Rad Laboratories Inc., Hercules, CA).

### Glycosidase Treatment and Velocity Sedimentation

Non-reducing sucrose gradient velocity sedimentation was performed on WT and *rom1* ^*-/-*^ retinal extracts as previously described [8, 13]. Retinal extracts (3 μg/sample for WT and 20 μg/sample for *rds* ^*C150S/C150S*^) or gradient fractions (9 μl/fraction) were denatured for ten minutes at 100°C using the denaturing buffer provided with the endoglycosidase H (EndoH)/PNGase F (New England Biolabs, Ipswich, MA) in a total reaction volume of 30 μl. This was then separated into 3 aliquots of 10 μl each and an additional 10 μl of enzyme master mix was added to each sample for a final reaction volume of 20 μl. One of the three samples received EndoH master mix (0.1 μl of EndoH plus manufacturer indicated buffer and water), the second sample received PNGaseF master mix (1 μl PNGaseF plus manufacturer indicated buffer, detergent, and water) and the third sample received mock master mix (EndoH buffer and water without enzyme). All samples were then incubated at 37°C for two hours. Experiments were repeated on 3–10 independent retinas/gradient fractions per group.

### Immunofluorescence (IF)

Transfected HEK293 cells were fixed with cold methanol:acetone (80%:20%) for 20 minutes. Cryosections were prepared from whole eyes fixed for 2h with 4% paraformaldehyde at 4°C collected at post-natal day 30 and underwent IF as described previously [10]. Briefly, after blocking in blocking buffer (1x PBS, pH 7.4, 1% fish gelatin, 2% donkey serum, 0.5% Triton X-100, 50 mg/ml bovine serum albumin), sections or cells were incubated overnight in primary antibodies in blocking buffer, followed by washing (4x 15 min with 1x PBS, pH 7.4), incubation in appropriate fluorescently labeled secondary antibodies in blocking buffer, additional washing, and mounting using ProlongGold mounting media (Life Technologies). Sections with a secondary antibody treatment only were used as a control to ensure the specificity of our antibodies and to normalize for background autofluorescence. All sections were treated with a 1% sodium borohydride solution for 2 min to minimize autofluorescence common in the retina. Cells and sections were imaged using an Olympus BX62 upright microscope equipped with a spinning disc confocal unit. Image analysis was performed using Slidebook v5 software (Intelligent Imaging Innovations, Denver, CO).

### Proximity Ligation Assay (PLA)

PLA assay was carried out using the Duolink® In Situ kit (Olink Bioscience, Uppsala, Sweden) on retinal cryosections. Antigen retrieval was carried out with methanol at -20°C for 20 minutes followed by several washing steps with water. The sections were treated with 1% sodium borohydride for 2 minutes to quench tissue autofluorescence, washed with water and 1x PBS and blocked for 90 minutes in blocking buffer (1xPBS, 1% fish gelatin, 2% donkey serum, 0.5% Triton X-100 and 5% bovine serum albumin) at room temperature. Primary antibodies were applied on sections in blocking buffer and incubated overnight at 4°C. After washing with 1x PBS, the secondary antibodies (anti-rabbit and anti-mouse) provided with the kit were diluted 1:5 in blocking buffer and incubated on the sections for one hour at 37°C. After washing with 1x PBS, ligation stock from the kit was diluted 1:5 in water, ligase was added (1:40 dilution) and incubated on the sections at 37°C for 30 minutes. Amplification stock from the kit was diluted 1:5 with water and polymerase was added at a dilution of 1:160 before application on the sections (washed with 1x Buffer A supplied with the kit after the ligation step) for 75 minutes at 37°C. After the final incubation step, sections are washed with 1x and 0.01x Buffer B from the kit subsequently. Sections were mounted and analyzed as described above for IF using filters for Texas Red. In each PLA experiment, paired sections underwent regular IF labeling using the identical protocol (except with standard fluorescent secondary antibodies rather than the PLA reagents) to confirm specificity of primary antibody labeling.

## Results

### Identification of Potential RDS/ROM-1 Interacting Partners

**Fig 1:**
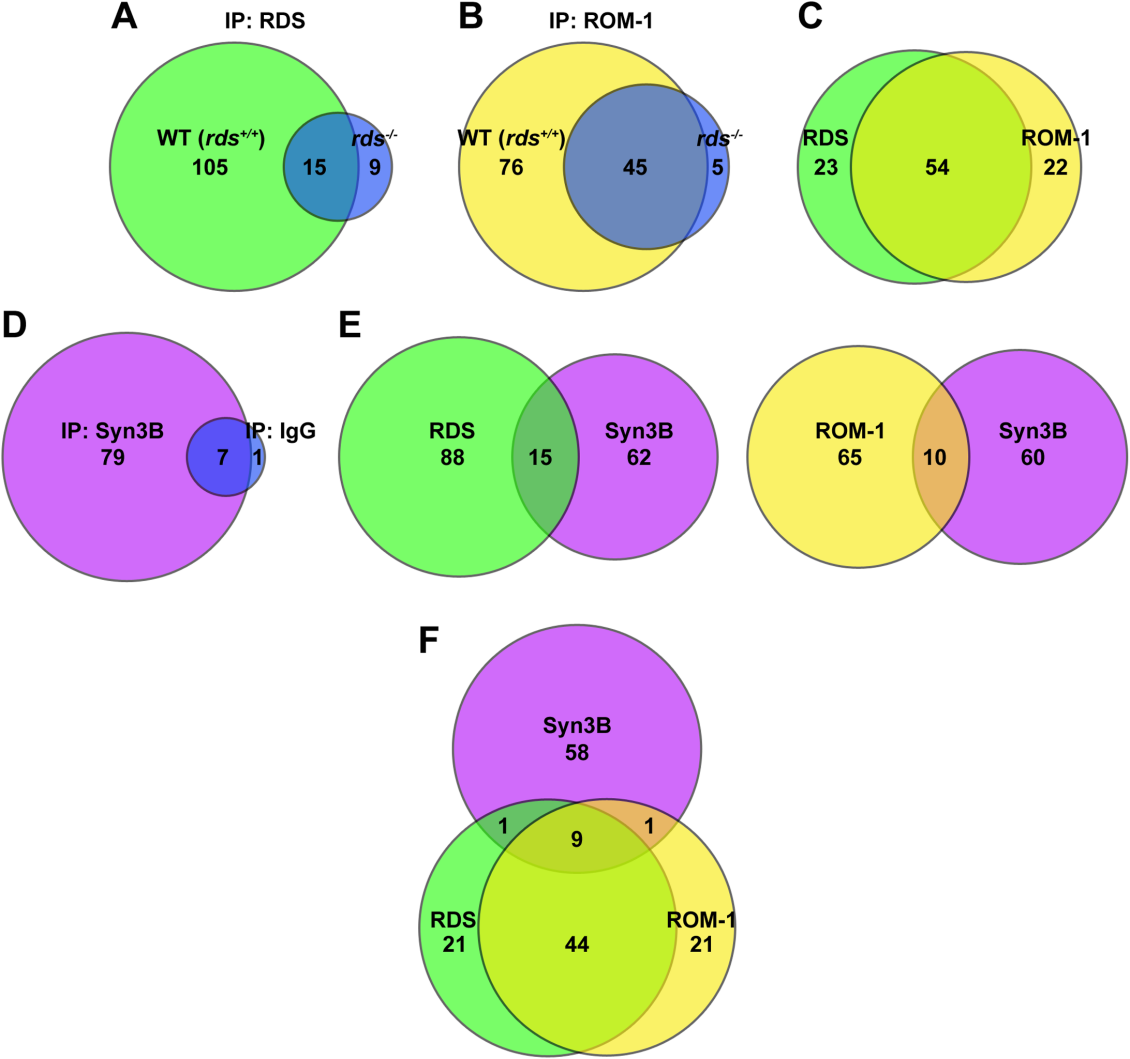
RDS/ROM-1 share interacting partners with each other and Syn3B. Euler diagrams depicting results from LC-MS studies to identify RDS/ROM-1 interacting partners. Numbers in each diagram represent proteins identified. **A-B.** WT and *rds* ^*-/-*^ retinal extracts were IPed using RDS mAB 2E7 (**A**) or ROM1 mAB 2H5 (**B**), cross-linked to sepharose followed by LC-MS. **C.** This diagram depicts overlap between proteins identified in RDS pull-down (**A**) and ROM-1 pull down (**B**) after subtraction of negative controls (blue circles **A-B**). **D.** WT retinal extracts were IPed with anti-Syn3B-SySy or IgG coupled to protein A beads followed by LC-MS. **E-F.** These diagrams depict overlap between proteins in the RDS (**E-left**) or ROM-1 (**E-right**) IP and the Syn3B IP, or overlap between all three (**F**).

Our goal was to isolate RDS complexes and identify novel RDS/ROM-1 interacting partners. For these studies, adult (postnatal day 30) WT mouse (Balb/C) retinal extracts were solubilized using CHAPS since, of the four detergents we tested, CHAPS was the mildest (to maximize identification of potential interacting partners) that still provided good RDS/ROM-1 solubilization (**S2 Fig**). LC-MS analysis was performed using sepharose columns cross-linked to RDS mAB 2E7 or ROM-1 mAB 2H5. As controls for the RDS and ROM-1 pulldowns, retinal extracts from *rds* ^*-/-*^ animals were used. As expected, RDS was identified in the ROM-1 IP and vice-versa (with high mascot scores). We also detected another known RDS binding partner, CNGβ [18], in the RDS IP. We began our assessment of novel interacting partners by eliminating known contaminants (such as keratins). Proteins identified in the *rds* ^*-/-*^ group from the RDS IP (blue, **Fig 1A**) were considered non-specific and were excluded. Although ROM-1 levels are very low in *rds* ^*-/-*^ retinas, there is some ROM-1 present, so proteins which appeared in the ROM-1 IP from *rds* ^*-/-*^ retinas (blue, **Fig 1B**) are not necessarily non-specific, but could be ROM-1 binding partners which do not require RDS. However, they were excluded from further analysis in this study as we were interested in components which interact with RDS or the RDS/ROM-1 complex. After manually removing proteins found in the negative controls by cross referencing the two lists, a substantial number of RDS and ROM-1 potential interacting partners remained (green and yellow **Fig 1A and 1B**), and of these, the majority (54 proteins) were common to the RDS and ROM-1 groups (**Fig 1C**).

Of the 54 proteins common to the RDS and ROM-1 groups, several were either SNARE or SNARE-associated proteins or other proteins potentially involved in protein trafficking (**Table 2**). SNAREs are known to play a role in targeting other proteins from the inner segment to the OS, so for this study, we narrowed our focus to this category. Specifically, two proteins identified by mass spectrometry (MS), Syntaxin 3 B (Syn3B) and synaptosomal-associated protein 25 (SNAP-25) are known to be involved in OS targeting of rhodopsin [28]. Syntaxin 3 has four different isoforms (A-D), but only isoform B is expressed in the mouse retina [29].

**Table 2:** SNARE and Trafficking Telated Proteins Identified in MS Analysis.

				Mascot Score		
Protein name	Protein ID	Mass in kDa	Max total sequence coverage	IP: RDS	IP: ROM1	IP: Syn3B
Syntaxin-binding protein 1 isoform a (Stxbp1, Munc18-1)	NP_001107041.1	69	19%	371	202	147
V-type proton ATPase 116 kDa subunit (VPP1)	NP_058616.1	96	12%	164	256	
V-type proton ATPase subunit d 1 (Atp6v0d1)	NP_038505	40	8%	125	161	246
V-type proton ATPase subunit E 1 (Atp6v1e1)	NP_031536	26	12%	187	206	96
V-type proton ATPase catalytic subunit A (Atp6v1a)	NP_031534	68	7%	81	65	
vesicle-fusing ATPase (N-ethylmaleimide sensitive factor, NSF)	NP_032766	83	5%	66	109	
synaptic vesicle glycoprotein 2A (SV2A)	NP_071313	83	9%	131	107	
ras-related protein Rab-1B	NP_083852	22	27%	182	121	
ras-related protein Rab-3A	NP_033027	25	25%	146	106	
ras-related protein Rab-2A	NP_067493	24	13%	102		
synaptotagmin-1 isoform 2	NP_001239271	47	26%	374	351	
vesicle-associated membrane protein-associated protein A	NP_038961	28	12%		118	153
Vesicle transport through interaction with t-SNAREs homolog 1B	NP_058080.2	27	14%			86
Syntaxin 1B	NP_077725.1	33	21%		114	213
Syntaxin 6	NP_067408	29	9%			76
Syntaxin 12	NP_598648	31	33%			231
Synaptoporin	NP_082328	31	15%			126
Synaptophysin	NP_033331.2	33	13%			83
Vesicle-associated membrane protein 2 (Vamp2)	NP_033523.1	13	35%	303	219	1104
synaptosomal-associated protein 25 (SNAP25)	NP_035558	23	15%	63		465
Syntaxin-3 isoform B (Stx3)	NP_001020478.1	33	8%	77	89	781

Syn3B was selected as the first candidate for further study. We performed an IP on CHAPS-extracted WT retinas using an antibody against Syn3B followed by LC-MS analysis as described above. Knock-out animals for Syn3B are not available, so we used pull-down with non-specific IgG as a negative control (**Fig 1D**). The IP with the Syn3B antibody pulled down a numerous proteins including several expected trafficking proteins as well as ROM-1 and RDS, thus providing initial confirmation of an interaction between RDS/ROM-1 and Syn3B. Multiple proteins overlapped between RDS/Syn3B and ROM-1/Syn3B pull-downs (**Fig 1E**) and a few proteins were present in all three (**Fig 1F**). All trafficking proteins found in one of the IPs (RDS/ROM-1/Syn3B) are summarized in **Table 2**.

### Interaction of RDS and ROM-1 with Syn3B

**Fig 2:**
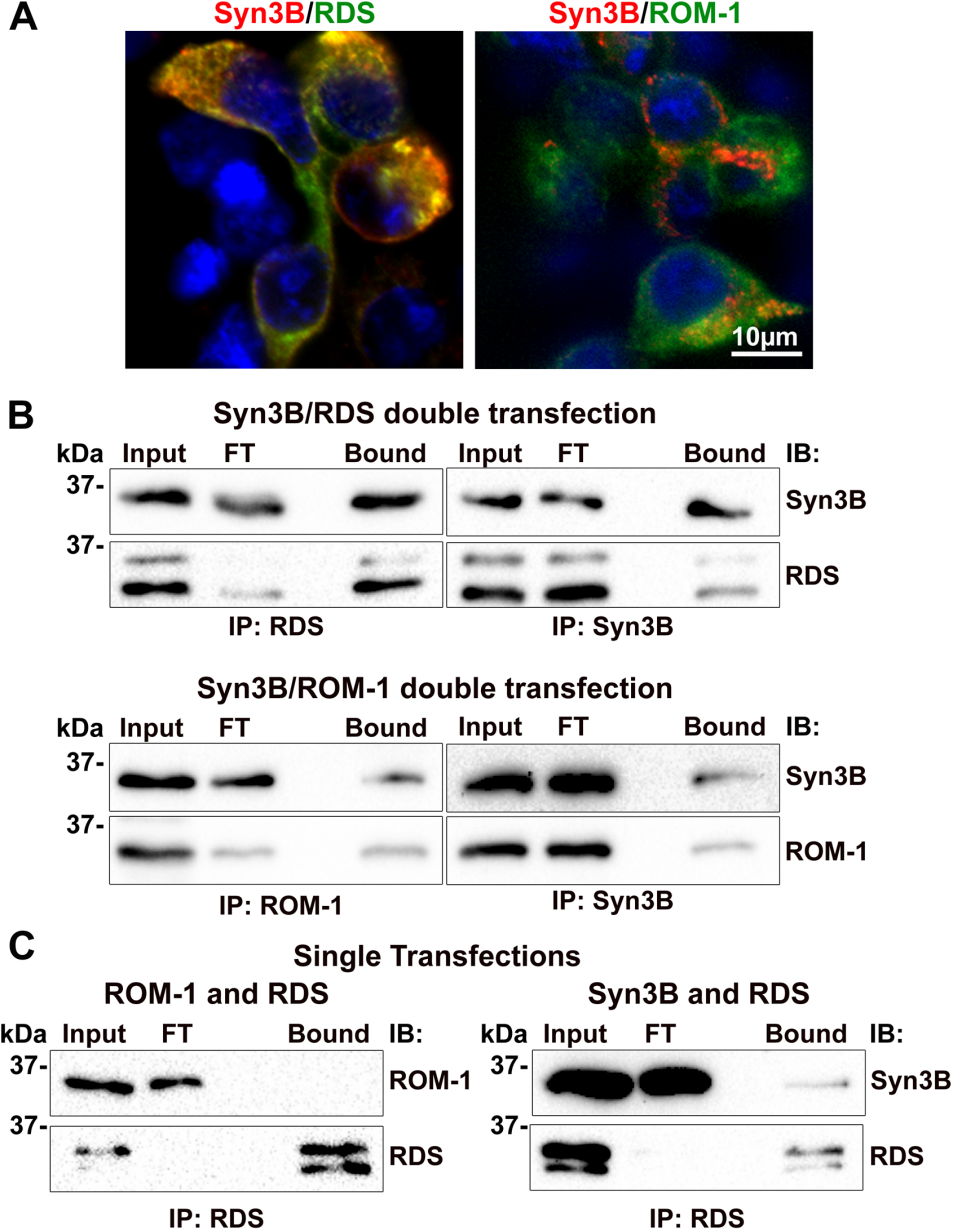
RDS and ROM-1 interact *in vitro* with Syn3B. **A-B**. HEK293 cells were double transfected with RDS/Syn3B and ROM-1/Syn3B. **A.** IF of transfected HEK293 cells demonstrating double transfection; RDS (RDS-CT) and ROM-1 (ROM1-CT) antibodies are shown in green, with Syn3B (mAB 12E5) antibody in red. **B.** Transfected cell extracts underwent reciprocal co-IP with RDS (RDS-CT), ROM-1 (mAB 2H5), or Syn3B (Syn3B-478) antibodies, and resulting WBs were probed for RDS (mAB 2B7), ROM-1 (mAB 2H5), or Syn3B (mAB 12E5) antibodies. **C.** HEK293 cells were single transfected with the indicated plasmids and cell extracts were subsequently mixed in a 1:1 ratio prior to IP (same antibodies as in **B**). FT: flow-through.

To begin our validation of interactions between RDS/ROM-1 and Syn3B, we conducted reciprocal co-IP studies using transiently transfected HEK293 cells (which do not endogenously express any of the proteins of interest). To facilitate detection of Syn3B along with RDS, we generated and characterized a new monoclonal antibody (mAB 12E5) against Syn3B (**S1 Fig**). This antibody gave a single band of the expected size in mouse and bovine retinal extracts and in transiently transfected Syn3B in HEK293 cells (**S1 Fig**, left). No band was detected in untransfected HEK293 cells, and all bands were indistinguishable from those obtained with commercially available antibodies for Syn3B (**S1 Fig**, right). To study RDS/ROM-1/Syn3B interactions, cells were co-transfected with vectors to express RDS and Syn3B or ROM-1 and Syn3B. Transfection efficiency was relatively high (~60–70%) and immunocytochemistry confirmed that RDS/Syn3B and ROM-1/Syn3B were usually co-expressed (**Fig 2A**) in the double transfected cells. After extraction with Triton-X 100, RDS and ROM-1 were both detectable in the bound fraction of the respective Syn3B IPs (**Fig 2B**, right panels) suggesting a direct interaction between RDS and Syn3B as well as ROM-1 and Syn3B. This interaction was confirmed by the reciprocal pull-down with RDS and ROM-1 (**Fig 2B**, left panels).

We have previously observed that RDS and ROM-1 do not form interactions when mixed together post-extraction; a finding we attributed to the early assembly of RDS/ROM-1 complexes in the ER and the fact that RDS/ROM-1 complexes occur within the same membrane. In contrast, stable fusogenic SNARE complexes can form even in cell free systems [30, 31] or post-extraction, consistent with the fact that these complexes are continually assembled and disassembled in multiple distinct cellular compartments. To determine which category the Syn3B/RDS interactions fell into, we single transfected HEK293 cells with RDS, ROM-1, or Syn3B. Extracts from these cells were then mixed in a 1:1 ratio (based on total protein) and subsequently IPed. As expected, RDS and ROM-1 failed to interact under these conditions (**Fig 2C**, left), while Syn3B and RDS did interact (**Fig 2C**, right). This suggests that RDS/Syn3B likely do not initially (i.e. at the time of synthesis) assemble into a complex, but rather that the RDS/Syn3B interaction occurs later.

**Fig 3:**
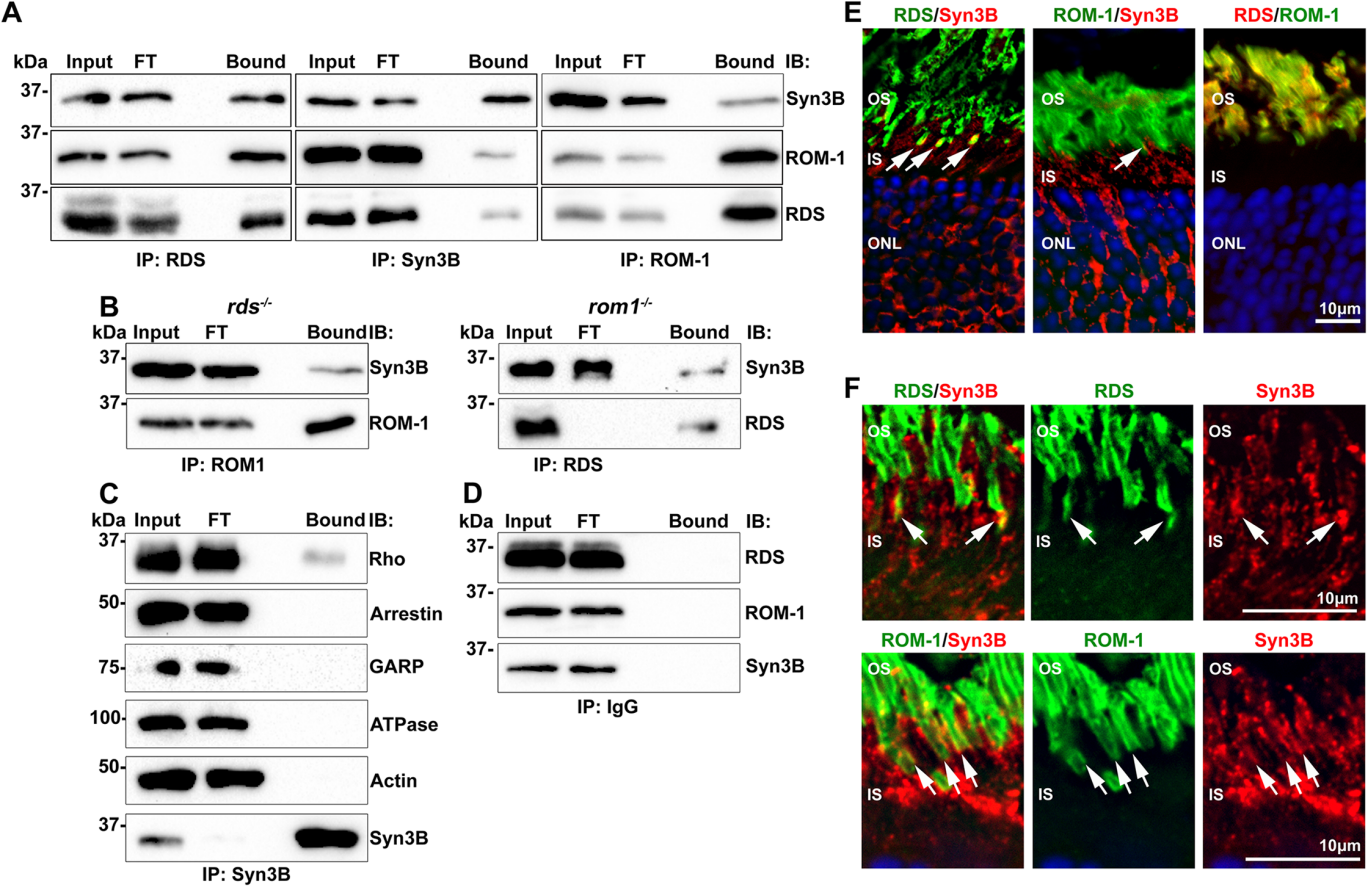
RDS and ROM-1 interact *in vivo* with Syn3B. **A.** Retinal extract from WT mice underwent reciprocal co-IP for RDS (RDS-CT), ROM-1 (mAB 2H5), and Syn3B (Syn3B-478 [22]) antibodies, and WB analysis was used to detect binding partners (RDS, mAB 2B7, ROM-1, mAB 2H5, Syn3B, mAB 12E5). **B.** IPs were performed using ROM-1 mAB 2H5 and RDS-CT antibodies from retinal extract from *rds* ^*-/-*^ and *rom1* ^*-/-*^ mice, respectively. Blots were probed for RDS (RDS-CT), ROM-1 (mAb 2H5) and Syn3B (mAb 12E5) antibodies. **C.** IP with anti-Syn3B (mAB 12E5) was performed on WT retinal extract and WBs were probed with antibodies against rhodopsin (Rho), arrestin 1, GARP, Na^+^/K^+^-ATPase (ATPase) and actin. None of the tested proteins except rhodopsin was detected after the pull down with Syn3B. **D** To ensure specificity of interactions, WT retinal extracts underwent IP with protein A beads and rabbit IgG. Resulting blots were probed for RDS (mAB 2E7), ROM-1 (mAB 2H5), and Syn3B (mAB 12E5). FT: flow through. **E.** Distribution of RDS (RDS-CT, green), ROM-1 (ROM1-CT, green middle, mAB 2H5, red right), and Syn3B (mAB 12E5, red) in the outer retina was assessed by IF, arrows show regions of co-localization. **F.** Frozen retinal sections from at least 3 different WT eyes were co-labeled with RDS or ROM-1 (RDS-PCT1, ROM-1 mAB2H5, respectively) in green, and Syn3B (Syn3B-529) in red and DAPI in blue and imaged at 100x. Shown are single planes from a confocal stack. Arrows indicate regions of co-localization. Scale bar: 10 μm. OS: outer segment, IS: inner segment, ONL: outer nuclear layer.

The expression of proteins in cell culture to test their interaction is convenient, enabling testing of individual pairs of proteins, but the setting is artificial. Overexpression of proteins in culture can induce aggregation, and neither HEK293 cells nor any other tissue culture system form elaborate structures like the OSs. Therefore, we next confirmed the interactions between Syn3B/RDS and Syn3B/ROM-1 *in vivo* by using Triton-X 100 extracted WT retinas for the IPs. The results closely mirrored the findings from the *in vitro* approach; Syn3B pulls down both RDS and ROM-1 and vice-versa (**Fig 3A**). Interestingly, in both *in vitro* and *in vivo* experiments, only a small fraction of RDS and ROM-1 interacts with Syn3B. Direct binding between RDS/Syn3B and ROM-1/Syn3B was confirmed *in vivo* using retinal extracts from *rds* ^*-/-*^ and *rom1* ^*-/-*^ animals (**Fig 3B**). To confirm that this binding was not due to non-specific interactions, we assessed a panel of positive and negative control proteins. In IPs with Syn3B, only rhodopsin (assessed using monoclonal antibody 1D4, [23], and known to be trafficked via a Syn3B-mediated pathway [20]) was detected in the bound fraction while several other OS (GARP, arrestin 1) and IS/cell body (actin, Na^+^K^+^ATPase) proteins were detected only in the input and flow through (**Fig 3C**). Similarly, rabbit and mouse IgG were used as negative controls, and no Syn3B, ROM-1, or RDS was detected in the bound fraction with IgG pull-down (**Fig 3D** shows a representative pull-down with rabbit IgG).

Consistent with previous findings, Syn3B is localized to the IS/cell body/synaptic layer (red, **Fig 3E**, left and middle, synaptic layer not shown) [32], while most RDS and ROM-1 reside in the OS. Interestingly, although RDS and ROM-1 are known to be synthesized in the IS and present there for the duration of time needed to traffic to the OS, they are not routinely detected in this compartment by us or others, [1, 21] with antibodies against either intracellular or extracellular portions of the protein. This is likely due to the large quantity of RDS/ROM-1 in the OS vs. the relatively small amount being synthesized at any given time in the IS. Thus a large region of co-localization visible by immunofluorescence between RDS/ROM-1 and Syn3B in the IS would not be expected. However, we do observe a small region of overlap between Syn3B and RDS (**Fig 3E and 3F** yellow, arrows) in the apical IS. This region of co-localization can be observed more clearly at 100x; the right panel of **Fig 3F** shows single confocal plane from a section co-labeled with RDS or ROM-1 (green) and Syn3B (red). We do not observe a region of Syn3B/RDS co-labeling in all cells, suggesting the amount of overlap between RDS and Syn3B varies from cell-to-cell and may depend on how much protein trafficking is occurring in any given cell. In addition, since most RDS/ROM-1 synthesized in the IS is not detected by standard IF, other approaches are needed to assess the extent of the interaction.

**Fig 4:**
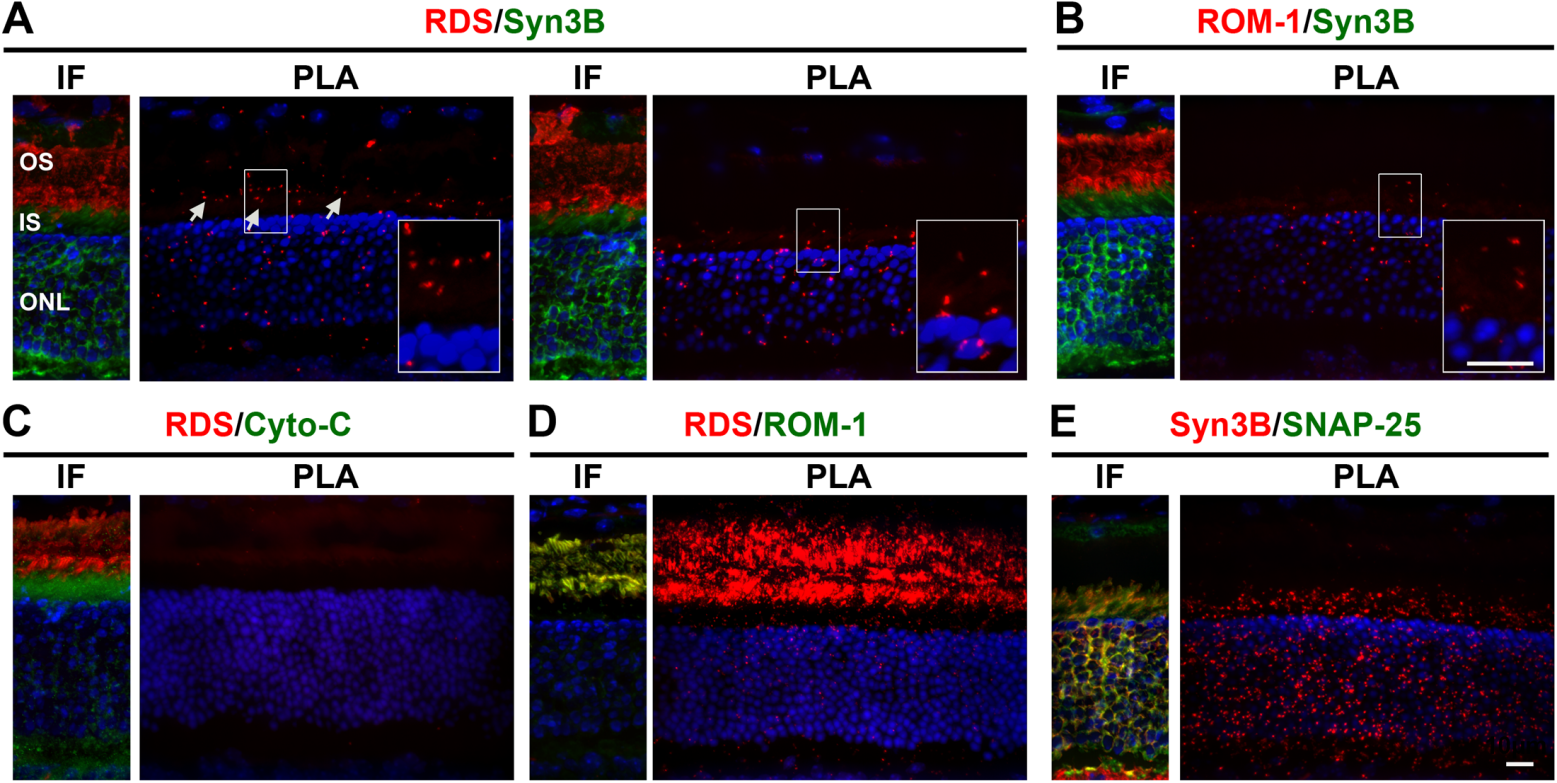
RDS/ROM-1/Syn3B interactions are visualized via PLA. Paired WT retinal cryosections underwent IF (left picture in each pair) or PLA (right picture in each pair) for the indicated proteins. Label colors correspond with colors in IF. Red labeling in PLA indicates interaction between the two proteins. Nuclei were stained with DAPI (blue). **A-B**. RDS/Syn3B (**A**) and ROM-1/Syn3B (**B**) interactions were assessed using RDS mAB 2E7, ROM-1 mAB 2H5, and Syn3B-478 antibodies. Insets show magnified regions. **C-E**. RDS/Cytochrome C (RDS mAB 2E7, Cyto-C polyclonal) served as a negative control (**C**) while positive controls included RDS/ROM-1 [RDS mAB 2E7, ROM1-CT antibodies, (**D**)], and Syn3B/SNAP-25 [(Syn3B-478, SNAP-25 antibodies, (**E**)]. OS: outer segments, IS: inner segments, ONL: outer nuclear layer. Scale bars, 10 μm.

Thus we turned to an alternative cell biological approach to study RDS/Syn3B and ROM-1/Syn3b interactions *in vivo*: proximity ligation assay (PLA). This approach involves using secondary antibodies to which short oligonucleotides are conjugated. The subsequent ligation of a fluorescently labeled third oligonucleotide which binds only if the two labeled proteins are in very close proximity enables fluorescent detection of protein interacting partners [33]. Using cryosections from WT retinas, we performed paired experiments with PLA and IF. In **Fig 4**, each image pair shows IF on the left (to confirm that antibodies were working, with colors corresponding to the labels above each image) and a corresponding PLA image on the right. In the PLA image, if the proteins do not interact, no signal is detected, while red spots indicate areas in which the two interacting proteins are found in close proximity. When we labeled with RDS/Syn3B (**Fig 4A**, two representative images shown) substantial PLA signal was detected. Although PLA labeling was less prominent with ROM-1/Syn3B it was still present (**Fig 4B**). No signal was detected in the negative control (**Fig 4C**, PLA performed using RDS and cytochrome C, an unrelated IS protein). Positive controls included RDS/ROM-1 and Syn3B/SNAP-25 (**Fig 4D and 4E**). RDS/Syn3B PLA signal is often concentrated at the apical IS (corresponding to the periciliary region, arrows, Fig 4A), however signal is also detected in the rest of the IS and cell body. PLA labeling throughout the IS and cell body is also found with Syn3B/ROM-1 (**Fig 4B**) RDS/ROM-1 (**Fig 4D**), and Syn3B/SNAP-25 (**Fig 4E**). This observation is consistent with the idea Syn3B may interact with newly synthesized RDS and ROM-1 which are found in the IS.

### Interaction of RDS and ROM-1 with Other SNAREs

**Fig 5:**
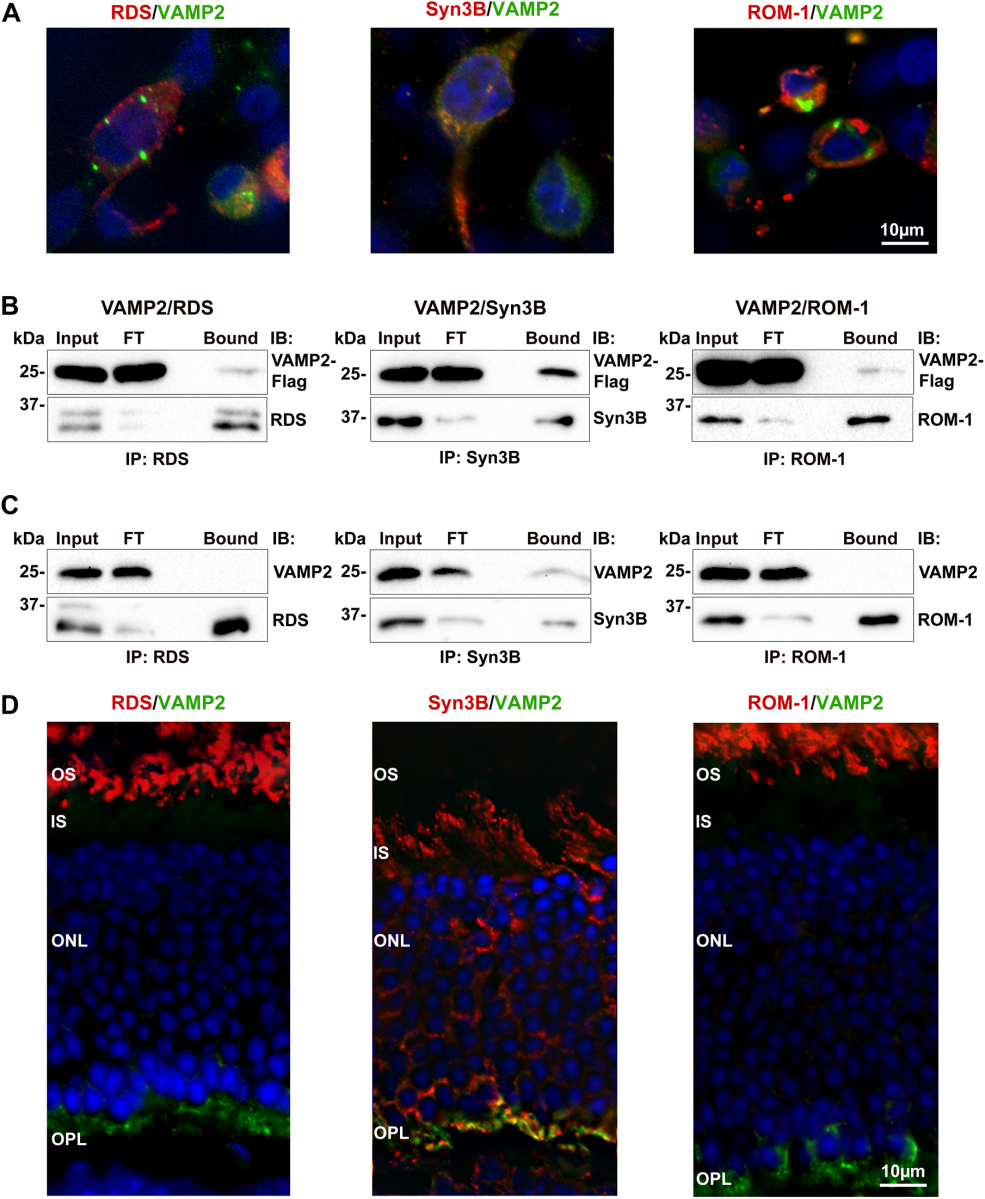
VAMP2 shows a weak interaction with ROM-1 and RDS *in vitro* but not *in vivo*. **A**. HEK293 cells were co-transfected with vectors containing RDS, ROM-1, or Syn3B and flag-tagged VAMP2 cDNA and fluorescently labeled for RDS (RDS-CT, red) ROM-1 (ROM1-CT, red), Syn3B (Syn3B-529, red), or VAMP2 (anti-DDK, green) antibodies. **B.** CHAPS-solubilized cell extracts were used for IP with RDS (RDS-CT), ROM-1 (mAB 2H5), and Syn3B (mAB 12E5) antibdoies, and resultant WBs were probed with RDS (mAB 2B7), ROM-1 (mAB 2H5), Syn3B (mAB 12E5), or VAMP2 (anti-DDK) antibodies. **C.** IP was performed on CHAPS-solubilized WT retinal extracts with the same antibodies as in **B**, and blots were probed for RDS (mAB 2B7), ROM-1 (mAB 2H5), Syn3B (mAB 12E5), and VAMP2 (VAMP2-SySy) antibodies. **D.** WT retinal sections underwent IF for VAMP2 (VAMP2-SySy, green), and RDS (RDS-CT, red), ROM-1 (ROM1-CT, red), or Syn3B (Syn3B-529, red) antibodies. Scale bars: 10 μm. OS: outer segment, IS: inner segment, ONL: outer nuclear layer. FT: flow through.

Having established that Syn3B interacts with RDS and ROM-1 we next examined other SNAREs expressed in photoreceptors and identified in our RDS proteomics screen, namely SNAP-25 and VAMP2. To validate potential interactions between RDS/ROM-1 and VAMP2, we double transfected HEK293 cells with RDS or ROM-1 and VAMP2 (**Fig 5A**). In preliminary IP experiments in which cell extracts were solubilized with Triton X-100, VAMP2 was not pulled down with RDS or ROM-1, but was abundant in the bound fraction after Syn3B pull-down. Syn3B binding to VAMP2 is essential for vesicle fusion in synapses so this latter result is expected [29]. Since our early experiments showed that the milder detergent CHAPS still solubilized RDS/ROM-1, we repeated the double-transfection experiment but used CHAPS to solubilize the proteins before the IP. Under these conditions, a small fraction of VAMP2 was pulled down with both RDS and ROM-1 (**Fig 5B**). This is consistent with the proteomics results, and suggests that binding of VAMP2 to RDS and ROM-1 is of a weaker nature than the binding of RDS/ROM-1 to Syn3B. However, when we repeated the IPs using retinal extracts, no VAMP2 was pulled down by RDS or ROM-1, even with solubilization in CHAPS (**Fig 5C**). Although some other proteomic studies have identified VAMP2 in OS preparations [34, 35], in distribution studies, both we and others detected VAMP2 primarily in the synaptic layer (**Fig 5D**, green) rather than in the IS or OS. Combined, these data suggest that VAMP2 likely does not directly bind RDS/ROM-1 in the retina.

**Fig 6:**
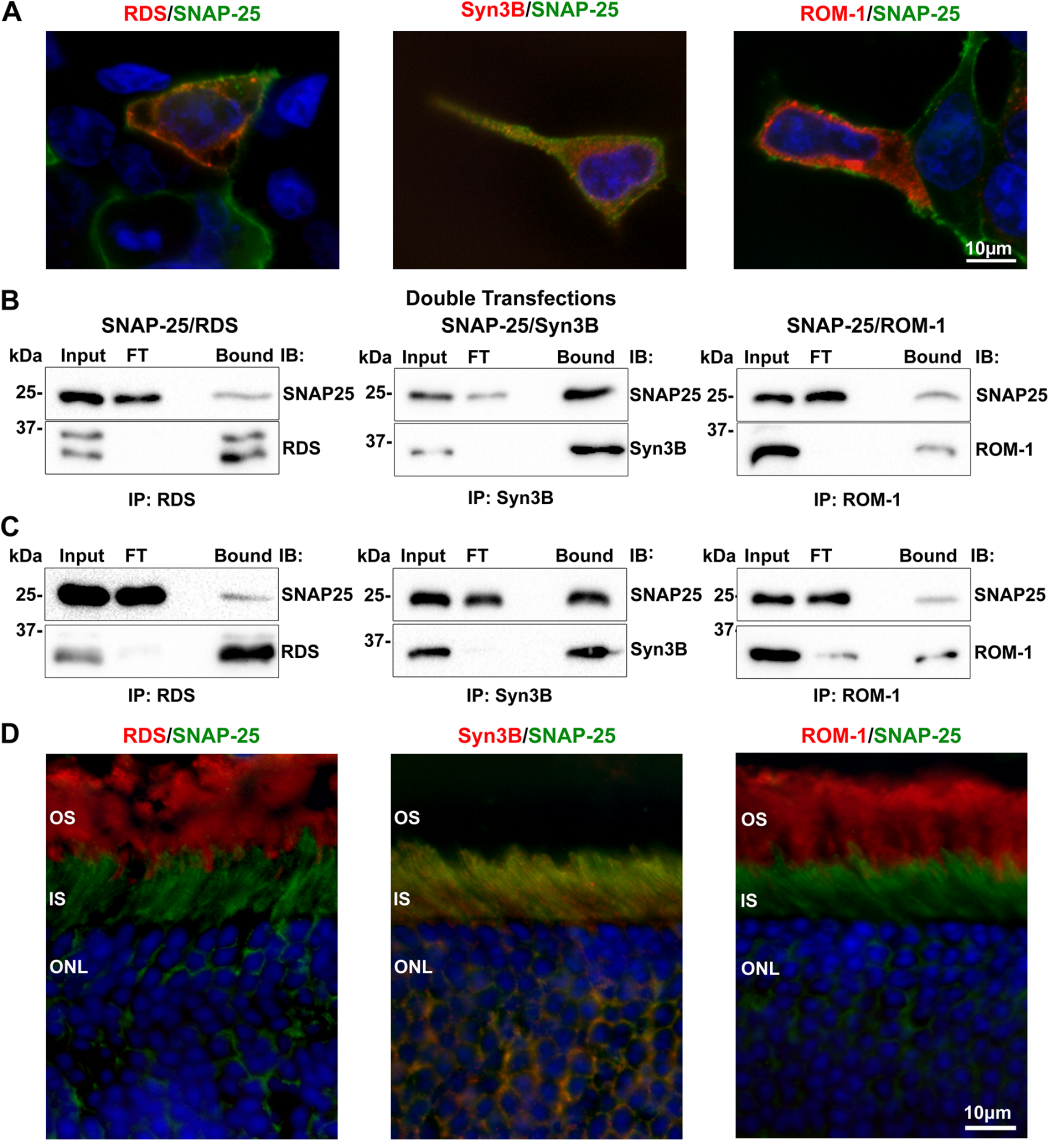
RDS and ROM-1 interact directly with SNAP-25 *in vitro* and *in vivo*. **A**. HEK293 cells were co-transfected with SNAP-25 (green) and either RDS (RDS-CT, red), ROM-1 (ROM1-CT, red) or Syn3B (Syn3B-529, red) antibodies. **B.** CHAPS-solubilized cell extracts underwent co-IP for RDS (RDS-CT), Syn3B (mAB 12E5), and ROM-1 (mAB 2H5) antibodies. Resulting blots were probed for SNAP-25, RDS (mAB 2B7), Syn3B (mAB 12E5), or ROM-1 (mAB 2H5) antibodies. **C.** CHAPS-solubilized WT retinal extract underwent IP and reducing SDS-PAGE/WB with the same antibodies as in **B**. **D.** WT retinal sections underwent IF to study distribution of SNAP-25 (green) in the outer retina in relation to labeling with RDS (RDS-CT, red), Syn3B (Syn3B-529, red), or ROM-1 (ROM1-CT, red) antibodies. Scale bars: 10 μm. OS: outer segment, IS: inner segment, ONL: outer nuclear layer. FT: flow through.

Finally, we assessed interactions between RDS/ROM-1 and SNAP-25, using both double transfected cells (**Fig 6A and 6B**) and WT retinas (**Fig 6C and 6D**). Cell extracts were solubilized in CHAPS and after IP with RDS, ROM-1, and Syn3B, SNAP-25 was detected in the bound fractions (**Fig 6B**), indicating that the interaction between RDS/ROM-1 and SNAP-25 is direct and not mediated by Syn3B (no endogenous Syn3B is detected in HEK293 cells). This interaction was also confirmed *in vivo*; IP of CHAPS extracted WT retinas with antibodies against RDS or ROM-1 showed SNAP-25 in the bound fraction (**Fig 6C**). From this we conclude that RDS and ROM-1 interact not only with Syn3B but also with SNAP-25. As expected, Syn3B and SNAP-25 also interacted *in vivo* (**Fig 6C**). In contrast to VAMP2, SNAP-25 (green, **Fig 6D**) co-localizes with Syn3B (red, middle **Fig 6D**) in the IS of photoreceptor cells and thus gives a similar distribution to Syn3B in reference to RDS (red, left **Fig 6D**) and ROM-1 (red, right **Fig 6D**). These cell biological and biochemical data are consistent with the idea that SNAP-25 interacts with newly synthesized RDS and ROM-1 in the IS.

### Syn3B Interacts with Multiple RDS/ROM-1 Complex Types

**Fig 7:**
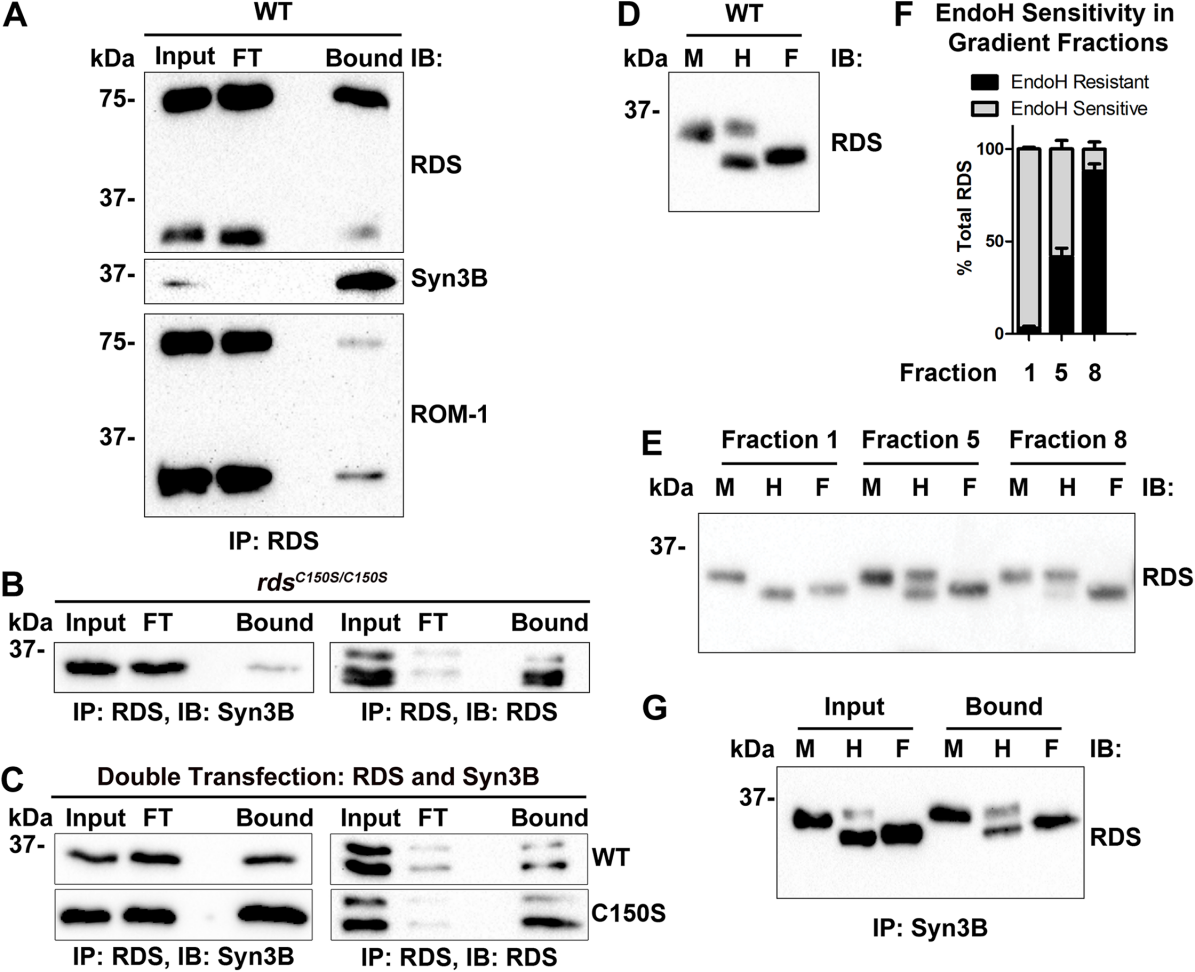
Syn3B associates with multiple types of RDS complexes. Protein extract from WT (**A**) or *rds* ^*C150S/C150S*^ (**B**) mouse retinas was IPed with anti-Syn3B-478. **A.** Elution/SDS-PAGE was performed under non-reducing conditions. RDS (mAB 2B7) and ROM-1 (mAB 2H5) antibodies show two bands on non-reducing western blot, monomers at around 37 kDa and dimers at around 75 kDa. **B.** Reducing SDS/PAGE after IP for RDS from *rds* ^*C150S/C150S*^ retinal extracts shows that oligomerization-incompetent RDS retains the ability to bind Syn3B. **C**. Extracts from HEK293 cells transfected with WT or C150S RDS and Syn3B underwent IP for RDS (using RDS mAB 2E7 or RDS-CT) antibodies. Blots were subsequently probed for RDS (RDS mAB 2B7) or Syn3B (mAB 12E5) antibodies. **D-G.** Total retinal extracts (**D**, WT N = 10, *rom1* ^*-/-*^ N = 6, *rds* ^*C150S/C150S*^ N = 3), fractions from non-reducing sucrose gradient velocity sedimentations (**E-F**), or retinal extracts from Syn3B IP (**G**) underwent digestion with EndoH (H), PNGase F (F), or were undigested (mock, M). Results from **F** are summarized graphically in **E**.

Having confirmed that RDS and ROM-1 interact with members of the SNARE family, we next asked what types of RDS/ROM-1 complexes are involved in these interactions. RDS and ROM-1 initially assemble into non-covalently linked homo- and hetero-tetramers with subsequent assembly into higher-order covalently linked complexes. These complexes are held together by C150-mediated disulfide bonds in the D2 loop of RDS [8, 9]. Multiple RDS/ROM-1 complex types are found in the OS: RDS/ROM-1 homo- and heterotetramers, RDS/ROM-1 homo- and heteromeric intermediate sized disulfide linked oligomers, and large RDS homooligomers, however, the different functions of these different complex types have not yet been elucidated. To understand which type of RDS/ROM-1 complex (covalent or non-covalently linked) interact with Syn3B, we IPed with Syn3B from WT retinal extract and eluted under non-reducing conditions. On non-reducing SDS-PAGE, the non-covalent RDS-RDS and RDS-ROM-1 interactions are interrupted by the SDS but the single C150-mediated intermolecular disulfide bond is preserved. As a result, under non-reducing conditions, intermediate and higher-order RDS complexes appear on western blots as a covalently bound dimer, while non-covalently linked complexes show up as monomers. RDS eluted from the Syn3B IP was both monomeric and dimeric (top, **Fig 7A**). Similarly, ROM-1 eluted from Syn3B IPs was both monomeric and dimeric (bottom, **Fig 7A**). To confirm covalent linkage was not required for interactions between RDS/Syn3B, we asked whether Syn3B could bind to C150S mutant RDS. This form of RDS lacks the cysteine which is responsible for the formation of disulfide bonds between RDS molecules, and as a result we and others have previously shown that it forms no covalently linked complexes, and thus no dimers are present on western blots prepared under either reducing or non-reducing conditions from either transgenic retinas or transfected cells [10, 11]. We conducted RDS IPs using knockin mice homozygous for the C150S mutation (*rds* ^*C150S/C150S*^, **Fig 7B**) or HEK293 cells transfected with C150S-RDS (**Fig 7C**). We find that C150S-RDS retains the ability to bind Syn3B (**Fig 7B and 7C**, bound) both *in vivo* and in tissue culture, confirming that assembly of RDS into covalently linked complexes is not required for RDS to bind to Syn3B.

### Syn3B Interacts with Conventionally and Unconventionally Trafficked RDS

Most transmembrane proteins which are synthesized in the IS traffic to the OS by a conventional secretory pathway, i.e. through the trans-Golgi. However, recent work has shown that RDS traffics through an unconventional pathway which bypasses the trans-Golgi [36]. Using a ciliated cell line, it was demonstrated that blocking trans-Golgi mediated trafficking did not block ciliary targeting of RDS, indicative of unconventional trafficking. Glycosylated proteins that bypass the Golgi (specifically the medial Golgi where Golgi mannosidase II resides) remain sensitive to endoglycosidase H (EndoH). Thus, EndoH sensitivity can be used as a marker for conventional vs. unconventional trafficking of OS proteins, provided the protein in question traffics properly to the OS and doesn’t accumulate in the ER (for example in cases of misfolded/degraded proteins). In keeping with this, RDS in both the cultured ciliated cells and xenopus/bovine retinal extracts exhibited robust EndoH sensitivity [36]. Because a small portion of RDS in the ciliated cells became EndoH resistant, suggesting a small fraction was processed via the conventional secretory pathway, we wanted to investigate whether Syn3B interacted with conventionally or unconventionally trafficked RDS.

First, we assessed what portion of RDS in the murine retina was trafficked via the conventional vs. unconventional pathway. WT retinal extracts underwent digest with EndoH (**Fig 7D,** lane E) or PNGaseF (**Fig 7D,** lane F, a positive control which removes all N-linked glycans) or were mock digested (i.e. included all steps except addition of the enzyme, **Fig 7D,** lane M) followed by western blotting. We find that 56.9% ± 5.5% of the total pool of RDS in the murine retina is EndoH sensitive, with the rest remaining resistant, suggesting that in the mouse retina some RDS traffics by both conventional and unconventional pathways. To ensure this outcome was not due to incomplete digestion, we conducted the experiments with increasing amounts of enzyme (up to 50x more than what was used in **Fig 7D**) without seeing any change in the fraction digested.

We next asked whether different types of RDS complexes had differential sensitivity to EndoH. WT retinal extracts underwent non-reducing velocity sedimentation on 5–20% sucrose gradients to separate RDS/ROM-1 complexes. Previously we have observed that covalently linked higher-order RDS homo-oligomers are found in fractions 1–3, intermediate sized RDS/ROM-1 heteromeric complexes (both covalent and non-covalently linked) are found in fractions 4–5 while heterotetramers are found in fractions 6–9 [8]. One gradient fraction representing each complex type (fractions 1, 5, and 8) then underwent glycosidase treatment (**Fig 7E and 7F**). We found that higher order RDS oligomers were completely EndoH sensitive, while intermediate complexes exhibited mixed EndoH sensitivity and tetrameric complexes were mostly EndoH resistant (**Fig 7E** and plotted in **Fig 7F**).

Finally, to determine whether Syn3B binds RDS trafficked through both the conventional and unconventional pathway, we conducted IP with Syn3B, and the eluents underwent EndoH treatment followed by western blotting for RDS. We find that Syn3B binds to both EndoH sensitive and EndoH resistant RDS (**Fig 7G,** bound lanes). This is consistent with our observation that Syn3B binds both covalently linked (largely unconventionally trafficked) and non-covalently linked (largely conventionally trafficked) RDS.

### Mutant Forms of RDS Associated with Abnormal Complex Formation Retain the Ability to Interact with Syn3B

**Fig 8:**
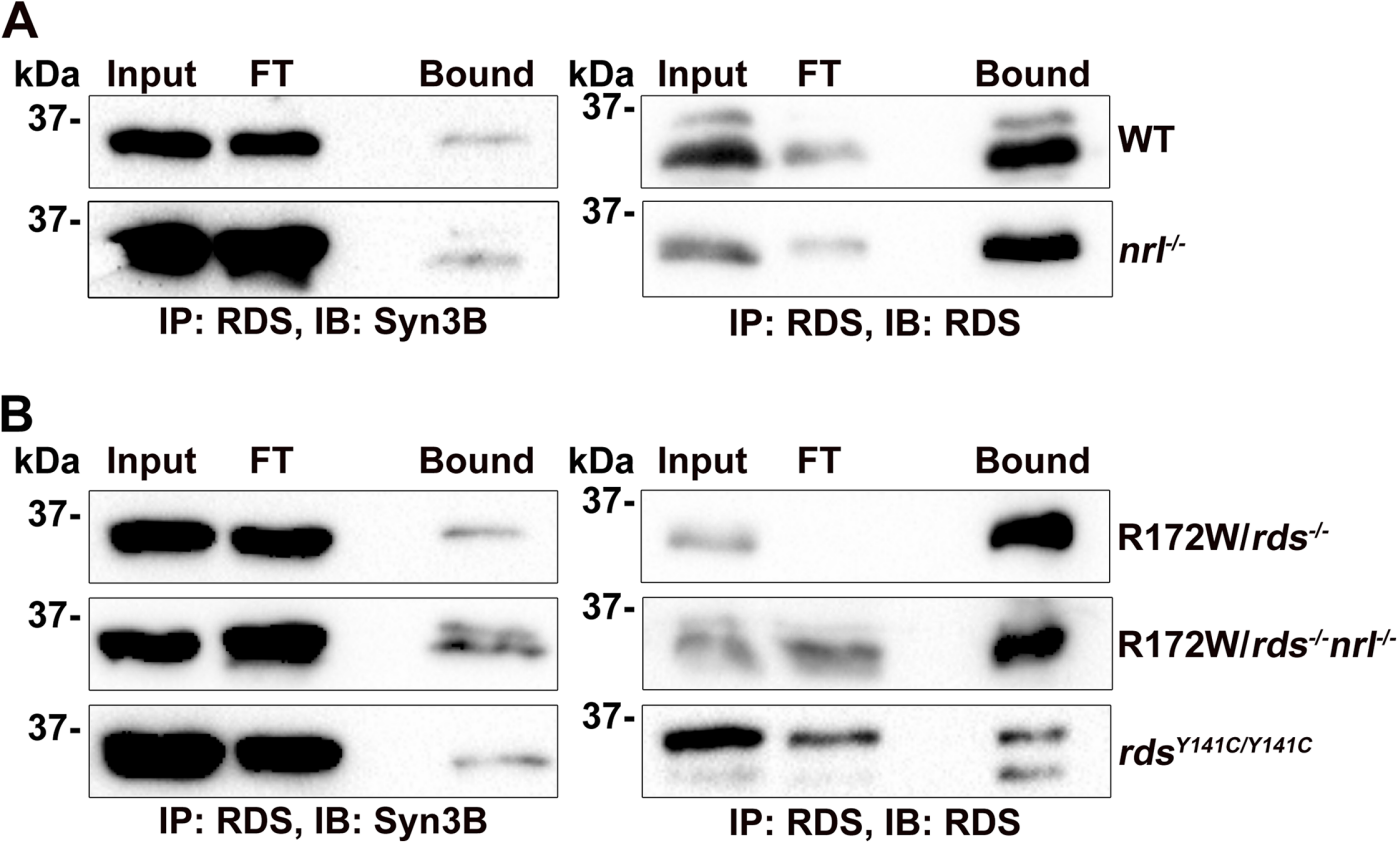
Mutant RDS that retains the ability to target to the OS interacts with Syn3B. **A-B.** Retinal extracts from WT and *nrl* ^*-/-*^ (**A**) or R172W transgenic and Y141C knockin (**B**) underwent IP for RDS (using RDS mAB 2E7 or RDS-CT) antibodies. Blots were subsequently probed for RDS (RDS mAB 2B7) or Syn3B (mAB 12E5) antibodies.

Various RDS mutations in patients lead to different forms of retinal degeneration which can affect rod- or cone-based vision (or both) and which can affect RDS/ROM-1 complex assembly. We have also previously demonstrated that rods and cones have a differential requirement for RDS and that some proteins such as the oligomerization-incompetent C150S protein, traffic differently in rods vs. cones [10, 14]. As one of our long-term goals is to identify binding partners that may contribute to these cell-type specific differences, we asked whether Syn3B could bind RDS in cones. The photoreceptors in the WT mouse retina are 95–97% rods, so we utilized retinal extracts from the *nrl* ^*-/-*^ knockout mouse retina (in which developing rods are converted to cone-like cells [37]). IP for RDS from the *nrl* ^*-/-*^ showed that RDS in cones retains the ability to bind Syn3B (**Fig 8A**).

We were also interested in the ability of Syn3B to interact RDS mutants which cause abnormal RDS/ROM-1 complex formation but not abnormal trafficking. The R172W mutation in RDS causes macular dystrophy [38] in patients and cone-dominant retinal degeneration in mice [13, 21]. The transgenic R172W protein traffics correctly to the OS and does not accumulate in the IS in spite of the fact that it induces abnormalities in ROM-1 complex formation [13]. We find that R172W-RDS retains the ability to bind Syn3B both on the rod (*rds* ^*-/-*^) and cone (*rds* ^*-/-*^ */nrl* ^*-/-*^) genetic backgrounds (top and middle, **Fig 8B**). We observe similar results with retinas from Y141C knockin mice (*rds* ^*Y141C/Y141C*^). The Y141C mutation causes pattern dystrophy in patients [39] and rod and cone degeneration in knockin mice [12]. Y141C protein forms highly abnormal covalently linked RDS/ROM-1 complexes, however it also traffics correctly to the OS and does not accumulate in the IS [12]. As in the case with R172W-RDS, Y141C-RDS retains the ability to bind Syn3B (bottom **Fig 8B**). These results suggest that though RDS/ROM-1 complexes assembly may be impaired to varying degrees by different RDS mutations, provided the abnormal protein complexes properly target to the OS, they retain the ability to bind Syn3B.

## Discussion

Here we use multiple methods to demonstrate that Syn3B and SNAP-25 are novel interacting partners for RDS and ROM-1. Syn3B and SNAP-25 are known to play a role in rhodopsin trafficking to the OSs [20]. Syn3B and SNAP-25 are located on the IS plasma membrane (in addition to the synaptic terminals) which is where the fusion of rhodopsin vesicles is thought to occur and the fusion of RDS- and ROM-1-bearing vesicles is predicted to occur. Given the localization of Syn3B and SNAP-25 (i.e. they are excluded from the OS) and their known role in trafficking and vesicle fusion, the most likely hypothesis to explain the role of Syn3B/RDS interactions is that Syn3B and SNAP-25 are involved in the fusion of RDS/ROM-1 containing vesicles in the IS. Experimental testing to elucidate the role of Syn3B/RDS interactions is a top priority, but must await the development of a retina-specific conditional Syn3B knockout mouse line since the global Syn3B knockout is embryonic lethal.

Syn3B is hypothesized to serve the same function for rhodopsin [20], i.e. mediating fusion of rhodopsin containing vesicles during OS targeting. Since rhodopsin vesicles are not thought to include RDS, it is possible that Syn3B-mediated vesicle fusion is a common component of many different trafficking pathways. This hypothesis is supported by our observation that Syn3B-bound RDS is both EndoH sensitive and EndoH resistant suggesting that both conventionally (i.e. like rhodopsin) and unconventionally trafficked RDS [36] bind SNAREs. Interestingly, we find that disease causing mutations which cause abnormalities in but do not abrogate complex formation do not impair the ability of RDS to interact with Syn3B. This suggests that while complex type may be a determinant of trafficking pathway, the type of complex formed (tetrameric/oligomeric/abnormal) is not critical for interactions between RDS and Syn3B. The observation that ROM-1 binds directly to Syn3B and SNAP-25 suggests that ROM-1 might be capable of traveling to the OS without RDS. Although initial studies suggested this was unlikely [40], more recent work has shown ROM-1 does not need RDS for OS targeting (although it cannot support OS formation without RDS) [41, 42].

Every SNARE complex consists of at least two members (and usually more), a t-SNARE on the target membrane and a v-SNARE on the membrane of the arriving vesicle. We identified Syn3B as a candidate t-SNARE however; the only v-SNARE in our LC-MS analysis was VAMP2. Although VAMP2 is in the retina and interacts with Syn3B [29, 34], it is not found in substantial amounts in the IS or OS [32]. A prior proteomics study identified VAMP2 in OS preparations [35], however fractionation methods for OSs have varying levels of purity and it is possible that VAMP2 might be contamination from another part of the retina, or might simply be present at very low levels. Given that we were unable to confirm the interaction between VAMP2 and RDS/ROM-1 *in vivo*, three formal possibilities exist. First, that VAMP2 is involved in RDS vesicle fusion, but does not bind to RDS or ROM-1 directly. This is possible since SNARE proteins do not necessarily bind to vesicular cargo; however the distribution of VAMP2 in the retina makes it unlikely. The second possibility is that another v-SNARE or VAMP is involved in RDS vesicle fusion, but that it also does not interact with RDS directly and thus did not appear in our LC-MS results. A third possibility is that Syn3B-mediated RDS vesicle fusion does not involve a v-SNARE. On the one hand, this seems unlikely; traditional SNARE-mediated vesicle fusion is well-characterized and requires both a t- and v-SNARE and RDS does not have a SNARE domain. However, RDS’ cytoplasmic C-terminal domain (already thought to be the site of OS targeting sequences [40]) is multi-functional, contains an amphipathic helix [5], and critically, the ability to mediate membrane fusion by unknown mechanisms [3, 4]. Interestingly, the ability of RDS to mediate fusion events requires assembly of RDS complexes, at least into tetramers, but possibly into larger covalently linked complexes, since addition of reducing agents or impairment of tetramer assembly prevents or retards fusion [4].

There is precedence for syntaxins to bind to non-SNARE partners involved in vesicular transport, including vesicular cargoes such as vesicle coat proteins, tethering factors, and Rab GTPases thought to be involved in regulating the targeting and docking of vesicles [43, 44]. In addition, some syntaxins have been shown to bind proteins not involved in vesicular transport such as ion channels, for example Syntaxin 1A binds to multiple subtypes of calcium channels in the plasma membrane, an interaction required for voltage sensitive neurotransmitter release [45, 46]. It is thus possible that direct binding between RDS and Syn3B/SNAP-25 means RDS may play a role in the actual fusion event or determination of tethering with the targeted membrane.

However, while it is possible that Syn3B-RDS interactions are a sign that Syn3B on the plasma membrane is involved in fusion of RDS-containing vesicles with that membrane, it is also possible that the interaction is a sign that the two proteins are actually trafficked together, i.e. that newly synthesized Syn3B is packaged into RDS-containing vesicles. Further testing of all these possibilities is required to understand the physiological roles of Syn3B/RDS/ROM-1 interactions and the role (if any) of RDS complex type in these processes.

Though our main goal in this study was to identify and verify novel RDS/ROM-1 binding partners, our work investigating whether these newly identified binding partners interacted with conventionally or unconventionally trafficked RDS brought to light interesting results regarding the trafficking of different types of RDS complexes. Previous work has shown that OS-depleted retinal extracts contain primarily RDS/ROM-1 tetramers, while OS-enriched retinal extracts contain primarily covalently-linked higher order RDS complexes [8]. This has led to the hypothesis that RDS assembles into tetramers in the IS and then traffics to the OS where some of it further oligomerizes into homomeric large covalently linked complexes while the rest remains in intermediate sized or tetrameric hetero-oligomers (with ROM-1). However, our data here showing that different types of RDS complexes are preferentially trafficked by the conventional vs. unconventional pathways indicate that the determination of RDS complex type (i.e. large covalently linked complexes vs. non-covalently linked tetramers) is made prior to or immediately after exit of newly synthesized RDS from the ER. This suggests that two distinct pools of RDS/ROM-1 are present from the time of initial synthesis, and that the final complex composition (i.e. determination of which RDS is fated to be part of large homooligomers vs. heterotetramers or intermediate complexes) is determined much earlier than the arrival of the proteins in the OS. Since disease causing mutations can drastically alter the assembly of RDS complexes, future work will likely involve investigation of the role of the different types of RDS complexes.

Widely varying blinding disease phenotypes are associated with point mutations in RDS, and these phenotypes have multiple different cellular/biochemical mechanisms [6, 47], many as yet poorly understood. Thus, identifying proteins that bind to RDS and thus have the potential to regulate its function, trafficking, or localization, is a critical goal. The trafficking of essential proteins to the photoreceptor outer segment is a crucial point in the development and maintenance of this specialized structure. In this study we are describing an interactions between RDS/ROM-1 and Syn3B and SNAP-25, two proteins well known for their function in vesicle fusion and protein trafficking. The same two proteins have been shown to be involved in the trafficking of rhodopsin and it is conceivable that they perform a similar function for RDS/ROM-1. It is therefore possible that disruptions in the transport of either rhodopsin or RDS/ROM-1 would have a detrimental influence on the trafficking of the others. Rhodopsin tends to mislocalize in a variety of retinal degeneration models, including the *rds* ^*-/-*^ [8, 41], and we have shown that in cones, opsins mislocalized with some forms of mutant RDS [10, 14]. Further exploration of the potential for abnormal trafficking to contribute to disease phenotypes will be a key part of studies designed to understand molecular mechanisms for degeneration and the development of novel therapies. The diversity of genes and mutations involved in retinal degeneration has always been a major hurdle for successful treatment. The discovery of common pathological pathways shared by different proteins and diseases, possibly including trafficking abnormalities, could simplify the development of new drugs and gene therapy vectors. Here we show that Syn3B and SNAP-25 bind multiple types of RDS/ROM-1 complexes, trafficked by both conventional and unconventional pathways. Elucidation of the functional role of these interactions will be a key next step as will evaluate additional RDS binding partners in rods vs. cones.

## Supporting Information

S1 Fig Characterization of monoclonal Syn3B antibody (12E5). Western blot incubated with the monoclonal Syn3B antibody shows a band of the expected size of Syn3B in mouse and bovine retinal extract, as well as in extracts from HEK293 cells transfected with a vector containing the cDNA sequence of Syn3B. There is no detectable Syn3B in untransfected HEK293 cells.
(TIF)

S2 Fig WT retinas were solubilized with the four detergents listed and supernatant (Sup) and pellet (Pel) fractions were separated by SDS-PAGE. Resultant WBs were probed for RDS (mAB 2B7) or ROM-1 (mAB 2H5) antibodies.
(TIF)
