# Supplementary figures and images for: SNAREs Interact with Retinal Degeneration Slow and Rod Outer Segment Membrane Protein-1 during Conventional and Unconventional Outer Segment Targeting
Source: PLoS One. 2015 Sep 25;10(9):e0138508. doi: 10.1371/journal.pone.0138508 (PMC4583372; doi:10.1371/journal.pone.0138508)

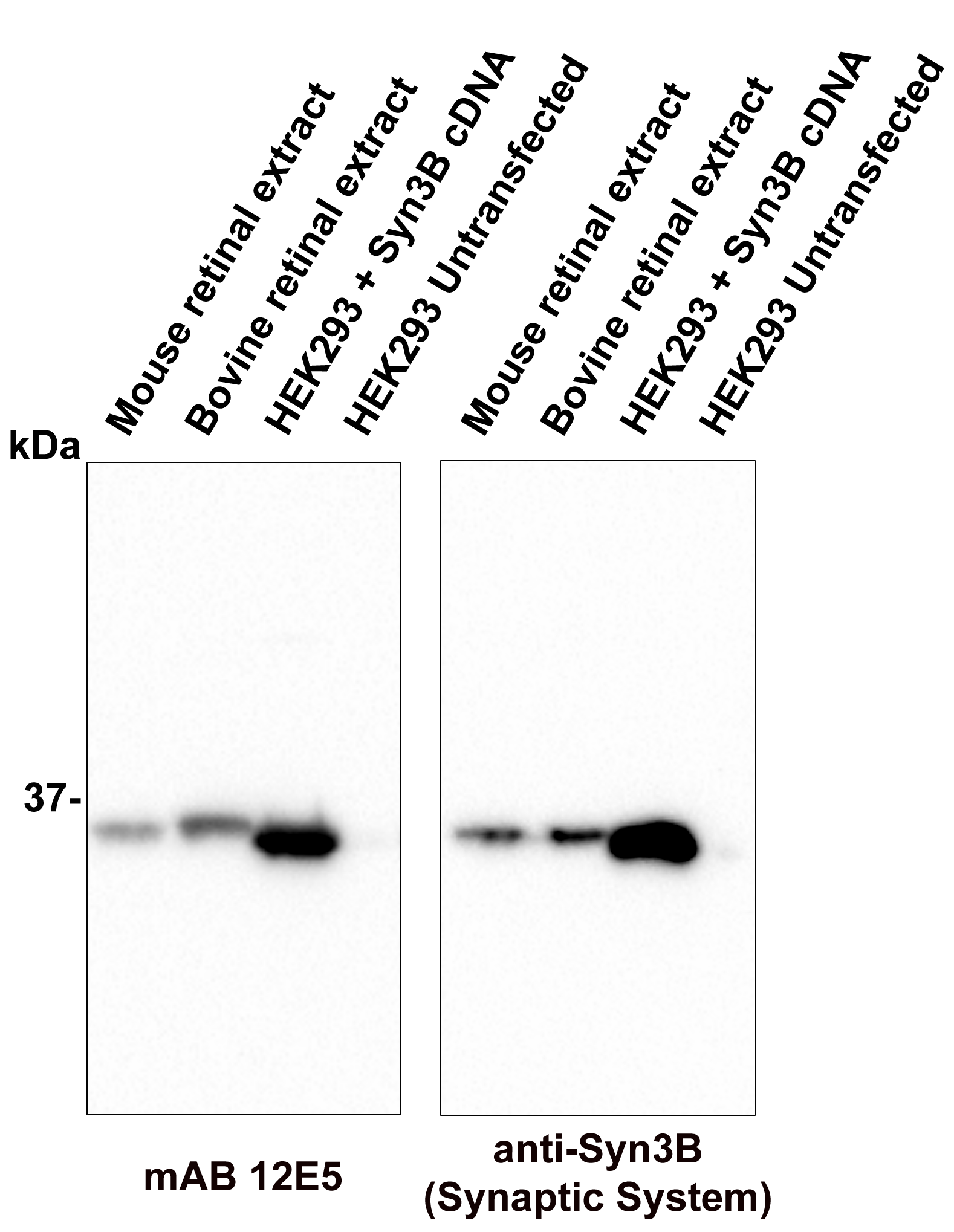

Supplement: S1 Fig — Western blot incubated with the monoclonal Syn3B antibody shows a band of the expected size of Syn3B in mouse and bovine retinal extract, as well as in extracts from HEK293 cells transfected with a vector containing the cDNA sequence of Syn3B. There is no detectable Syn3B in untransfected HEK293 cells. (TIF) [file pone.0138508.s001.tif]

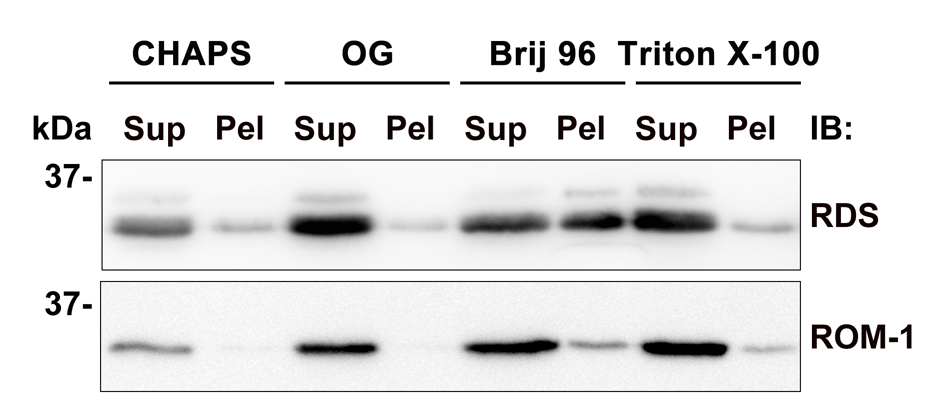

Supplement: S2 Fig — Resultant WBs were probed for RDS (mAB 2B7) or ROM-1 (mAB 2H5) antibodies. (TIF) [file pone.0138508.s002.tif]
